# More Feedback Is Better than Less: Learning a Novel Upper Limb Joint Coordination Pattern with Augmented Auditory Feedback

**DOI:** 10.3389/fnins.2016.00251

**Published:** 2016-06-06

**Authors:** Shinya Fujii, Tea Lulic, Joyce L. Chen

**Affiliations:** ^1^Canadian Partnership for Stroke Recovery, Sunnybrook Research InstituteToronto, ON, Canada; ^2^Graduate School of Education, The University of TokyoTokyo, Japan; ^3^Department of Kinesiology, McMaster UniversityHamilton, ON, Canada; ^4^Department of Physical Therapy and Rehabilitation Sciences Institute, University of TorontoToronto, ON, Canada

**Keywords:** motor learning, augmented feedback, guidance hypothesis, auditory feedback, sonification

## Abstract

Motor learning is a process whereby the acquisition of new skills occurs with practice, and can be influenced by the provision of feedback. An important question is what frequency of feedback facilitates motor learning. The guidance hypothesis assumes that the provision of less augmented feedback is better than more because a learner can use his/her own inherent feedback. However, it is unclear whether this hypothesis holds true for all types of augmented feedback, including for example sonified information about performance. Thus, we aimed to test what frequency of augmented sonified feedback facilitates the motor learning of a novel joint coordination pattern. Twenty healthy volunteers first reached to a target with their arm (baseline phase). We manipulated this baseline kinematic data for each individual to create a novel target joint coordination pattern. Participants then practiced to learn the novel target joint coordination pattern, receiving either feedback on every trial i.e., 100% feedback (*n* = 10), or every other trial, i.e., 50% feedback (*n* = 10; acquisition phase). We created a sonification system to provide the feedback. This feedback was a pure tone that varied in intensity in proportion to the error of the performed joint coordination relative to the target pattern. Thus, the auditory feedback contained information about performance in real-time (i.e., “concurrent, knowledge of performance feedback”). Participants performed the novel joint coordination pattern with no-feedback immediately after the acquisition phase (immediate retention phase), and on the next day (delayed retention phase). The root-mean squared error (RMSE) and variable error (VE) of joint coordination were significantly reduced during the acquisition phase in both 100 and 50% feedback groups. There was no significant difference in VE between the groups at immediate and delayed retention phases. However, at both these retention phases, the 100% feedback group showed significantly smaller RMSE than the 50% group. Thus, contrary to the guidance hypothesis, our findings suggest that the provision of more, concurrent knowledge of performance auditory feedback during the acquisition of a novel joint coordination pattern, may result in better skill retention.

## Introduction

Different joint coordination patterns can be used to achieve one specific motor action (Bernstein, 1967). For example, a person can reach for a cup of coffee on a table in front of them by extending the elbow and flexing the shoulder without moving the trunk. The same action can also be achieved by flexing the trunk with minimal or even no movement of the elbow and shoulder. Although in both cases, the goal of the movement is achieved, the biomechanical efficiency of the movements differs depending on how the joints are coordinated (Hirashima, 2011 for a review). Organized joint coordination patterns allow a person to use muscles efficiently and to prevent muscle fatigue (Furuya et al., 2009). Thus, achieving biomechanically and physiologically efficient movements requires the learning and execution of organized joint coordination patterns. The redundancy of joint coordination patterns can be an issue in motor rehabilitation as the restoration of normal joint coordination patterns is often a challenge for people with movement disorders (Levin, 1996; Cirstea and Levin, 2007). For example, individuals with stroke often implement compensatory strategies (i.e., reaching for a cup of coffee by bending forward with the trunk) given their impaired motor control. Although the movement goal is achieved, use of compensatory strategies may result in longer-term problems such as pain, discomfort, and joint contractures (Levin, 1996; Cirstea and Levin, 2007). Thus, improving movement quality through the re-learning of organized joint coordination patterns is of importance for people with movement disorders.

One strategy that can facilitate the motor (re)learning of organized joint coordination patterns is the use of *augmented feedback*. Augmented feedback is external information provided about the movement that is supplemental to *inherent feedback* (Schmidt and Lee, 2011). Inherent feedback is intrinsic sensory information that is naturally available to an individual during the movement (e.g., vision or proprioception of limbs). The provision of augmented feedback may be relevant when an individual is learning to execute a new skill such as the golf swing, or when a person with a stroke is re-learning how to reach for a cup of coffee. Augmented feedback can be classified into two types: *knowledge of results (KR)* and *knowledge of performance (KP)*. KR refers to feedback about the outcome of a movement, such as the score in a game of darts. KP refers to feedback about the nature of the movement pattern, such as whether the elbow was sufficiently extended when throwing a dart. Thus, KP feedback may be especially relevant if one wants to provide feedback about joint coordination patterns.

In a typical study that examines effects of augmented feedback on motor learning, participants first practice a task with augmented feedback during a period known as the *acquisition phase* (Schmidt and Lee, 2011). Performance during the acquisition phase is thought to represent a combination of effects derived from learning and the temporary guidance provided by augmented feedback. Therefore, to evaluate whether the skill has been learned, performance is tested during the *retention phase*, when the task is performed without augmented feedback. As such, acquisition and retention data are analyzed separately because the former may be conflated by the temporary guidance effect of augmented feedback. In contrast, the retention data more clearly represents the degree to which a skill has been learned and retained (Winstein and Schmidt, 1990; Nicholson and Schmidt, 1991; Vander Linden et al., 1993; Tal, 1995; Wulf et al., 1998, 2010; Park et al., 2000). The retention phase can be further subdivided into immediate and delayed retention phases (Winstein and Schmidt, 1990). Immediate retention evaluates performance without feedback, shortly after skill acquisition on the same day. Delayed retention evaluates performance without feedback, usually on the following day, or even after a longer period.

Several studies have investigated what type or frequency of augmented feedback facilitates the retention of a motor skill (Winstein and Schmidt, 1990; Nicholson and Schmidt, 1991; Vander Linden et al., 1993; Tal, 1995; Wulf et al., 1998; Park et al., 2000). However, a yet to be resolved question is with what frequency should feedback be provided to facilitate retention. The influential guidance hypothesis (Salmoni et al., 1984; Schmidt et al., 1989) postulates that too much feedback is detrimental to motor skill learning. The guidance hypothesis makes three assumptions. First, frequent feedback, such as its provision on every training trial, is assumed to negatively affect learning because the learner comes to rely on augmented feedback at the expense of using his/her own inherent feedback. This reliance leads to the deterioration of performance when the augmented feedback is unavailable during the retention test.

Second, the guidance hypothesis assumes that a reduced frequency of augmented feedback (e.g., providing feedback on every other training trial) may facilitate learning because it promotes the learner to use their own inherent feedback during the no-feedback trials (Salmoni et al., 1984; Schmidt et al., 1989). The no-feedback trials also provide the learner with the opportunity to integrate information from previous feedback trials, with information derived from their own inherent feedback systems. The active use of inherent feedback systems during the no-feedback trials may help the learner form a motor command to execute a target movement without relying on the augmented feedback (Salmoni et al., 1984; Schmidt et al., 1989). Thus, when performance of the skill is tested at retention, there is no/less deterioration in performance because the learner is not reliant on the augmented feedback.

Third, the guidance hypothesis assumes that frequent augmented feedback may also increase movement variability (Salmoni et al., 1984; Schmidt et al., 1989). Movement variability is thought to increase because frequent augmented feedback encourages the learner to over-correct the movement (the so-called, *maladaptive short-term corrections*) even when performance is relatively close to the target (Schmidt, 1991). Therefore, taken together, the guidance hypothesis postulates that a reduced frequency of augmented feedback facilitates motor learning.

To our knowledge, the guidance hypothesis, or the optimal frequency of feedback, has not been tested in the context where individuals learn movements with augmented KP auditory feedback, provided concurrently with performance. Concurrent KP auditory feedback may be relevant for learning joint coordination patterns, especially in people with movement disorders who may benefit from the re-learning of biomechanically and physiologically efficient movements. This is because feedback provided concurrently to the movement (as opposed to at the end, i.e., terminal feedback), may facilitate online motor planning of a joint coordination pattern. Furthermore, there may be a difference between the efficacy with which auditory and visual feedback facilitate motor learning. Auditory relative to visual feedback may guide movements in a temporally more efficient way given that the auditory system is generally better at resolving temporal information (Repp and Penel, 2002, 2004; Patel et al., 2005; Hove et al., 2010).

We developed our concurrent KP auditory feedback via the *sonification* of movements. Sonification refers to the use of sounds to convey information for the purposes of facilitating communication (Dubus and Bresin, 2013 for a review). For example, a sound variable such as loudness can be mapped onto a kinematic variable such as the vertical hand position. Here, the sound would get louder as the arm moves upwards, and quieter when the arm moves downwards. Recently, there has been increasing interest in understanding whether sonified feedback can facilitate motor (re)learning (Sigrist et al., 2013, 2015; Scholz et al., 2014, 2015). For example, a recent study mapped pitches of a violin sound onto oar movements for rowing, and showed that sonified feedback facilitates the learning of a target rowing velocity (Sigrist et al., 2015). Another study mapped pitch, brightness, and loudness of a synthesized sound onto the arm movements of stroke patients, in 3D space, and showed that re-training movements with sonified feedback improved arm motor functions (Scholz et al., 2015). However, there are at least two gaps in the literature that can be further explored to better understand the role of sonified feedback in motor learning. First, prior work discussed above sonified the endpoint movement. In the present study, we map a sound variable onto the error related to the performed joint coordination pattern. Thus, our novel work tests whether sonified feedback facilitates the motor (re)learning of joint coordination patterns. Second, these sonification studies compared the effect of sonified feedback with that of no feedback (Scholz et al., 2015) or visual and visuohaptic feedback (Sigrist et al., 2015). In the present study, we test the influencial guidance hypothesis to investigate with what frequency sonified feedback should be provided to facilitate motor (re)learning. In accordance with the guidance hypothesis we postulate a reduced frequency of sonified feedback results in better retention of a learned joint coordination pattern.

We developed a sonification system that delivered a 440-Hz pure tone sound, which varied in intensity, in proportion to the error in the joint coordination pattern relative to a target pattern. We compared motor learning of the novel joint coordination pattern in two groups of healthy participants; one that received feedback on every training trial [i.e., 100% auditory feedback (AF)], and one that received feedback on every other training trial (i.e., 50% AF). According to the guidance hypothesis, we predicted: (1) The 50% AF group would show better retention of the learned joint coordination pattern compared to the 100% AF group because a reduced frequency of feedback encourages individuals to use inherent feedback naturally available to them; (2) The 50% AF group would show less variable joint coordination patterns than the 100% AF group because a reduced frequency of feedback prevents individuals from making maladaptive corrections to their movement patterns.

## Methods

### Participants

Twenty right-handed healthy individuals (16 females and 4 males) with normal hearing and no history of neurological or musculoskeletal disorders participated in this study. Participants were randomly assigned to one of two groups: 100 or 50% AF (*n* = 10 each). Age, handedness as assessed by the Edinburgh Handedness Inventory (Oldfield, 1971), number of years of education, and number of years of musical training are summarized in Table 1. The study was approved by the Institutional Review Board of Sunnybrook Health Sciences Centre and all participants provided written informed consent. Participants were compensated for their time and transportation.

**Table 1:** **Participant demographics**.

**Group**	**Mean (*****SD*****)**		***P*-values**
	**50% (*n* = 10, 2 males)**	**100% (*n* = 10, 2 males)**	
Age (years)	33.9 (15.4)	34.0 (16.7)	0.631
Handedness^*^	89.0 (22.8)	92.0 (16.2)	1.000
Years of education	14.6 (3.8)	13.5 (2.8)	0.481
Years of musical training	7.0 (7.7)	8.4 (5.2)	0.739

* *Laterality quotient assessed by the Edinburgh Handedness Inventory (Oldfield, 1971). Associated p-values for results from the independent-samples Mann-Whitney U-tests comparing 50 and 100% auditory feedback groups*.

### Hearing tests

In this study, participants learned a novel upper limb joint coordination pattern (see Section “Reaching Task” below) with augmented auditory feedback. The feedback was a sound that varied in intensity in proportion to the error of the joint coordination pattern relative to a target pattern. Therefore, to ensure participants were able to perceive these sounds, they underwent two hearing tests prior to the reaching task. A 440-Hz pure tone was used as the sound stimulus. The sounds were created with custom written C++ scripts and outputted as an analog signal with the analog input/output (AIO) board (ADA16-32/2CBF, Contec Co., Ltd., Japan). The analog signal was amplified by speakers (MM-SPWD2SV, Sanwa, Japan) and the sound was delivered to participants via headphones binaurally (MDR-NCB, SONY, Japan).

#### Hearing test #1: Assessment of hearing threshold

This test determined the loudness threshold at which a participant heard a tone. This ensured all participants were able to perceptually hear the sounds, and ensured sound intensities were perceptually-equated across participants.

**Figure 1:**
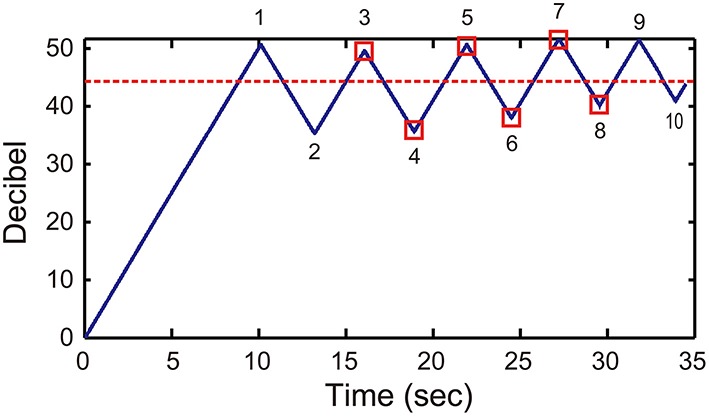
**Hearing test #1**. An example audiogram obtained from self-recording audiometry. The vertical axis denotes the intensity of the 440-Hz pure tone in decibels relative to the intensity at the beginning of the trial. The turnaround points marked by 1, 3, 5, 7, and 9 denote the times during which a participant pressed a switch indicating they heard the tone; turnaround points 2, 4, 6, 8, and 10 denote the times during which the participant released the switch indicating they did not hear the tone. The hearing threshold (the dashed line) was calculated as the average of the middle six turnaround points, marked by open squares.

To determine the hearing threshold, we used the Bekesy audiometry method, which is a self-recording audiometer (Bekesy, 1947). In this method, an increase or decrease in sound intensity is controlled by the action of a switch that participants press on. When the switch is pressed, sound intensity begins to decrease. Once the participant no longer perceptually hears the tone, he/she releases the switch. At this point, the sound intensity begins to increase and when the participant perceptually hears the tone again, he/she presses the switch (see Figure 1). Therefore, by asking participants to press the switch when they hear the tone and to release the switch when they no longer hear the tone, an individual's hearing threshold can be determined via this self-recording approach (Bekesy, 1947).

The sound intensity of a 440-Hz pure tone either increased or decreased at a rate of 5 decibels (dBs) per second. One trial consists of 10 time-points at which the switch was pressed on and off (see the numbered turnaround points in Figure 1). The hearing threshold in a trial was calculated as the average of the middle six time points (see the turnaround points marked by red squares in Figure 1). Thus, we discarded two time points at the beginning and at the end, and only analyzed stable responses. Participants practiced until they became familiar with the task. After practice, four trials were recorded from which the mean hearing threshold was calculated.

#### Hearing test #2: Detection of a change in sound intensity

This test determined the perceptual threshold at which a participant detected a change in the sound intensity of a 440-Hz pure tone. In this study, sound intensity was manipulated such that the louder the sound, the larger the joint coordination error during reaching (see Section “Creation of Auditory Feedback” below). Therefore, this test ensured participants could perceptually detect changes in sound intensity so that they could use this information to minimize their joint coordination error during the reaching task.

**Figure 2:**
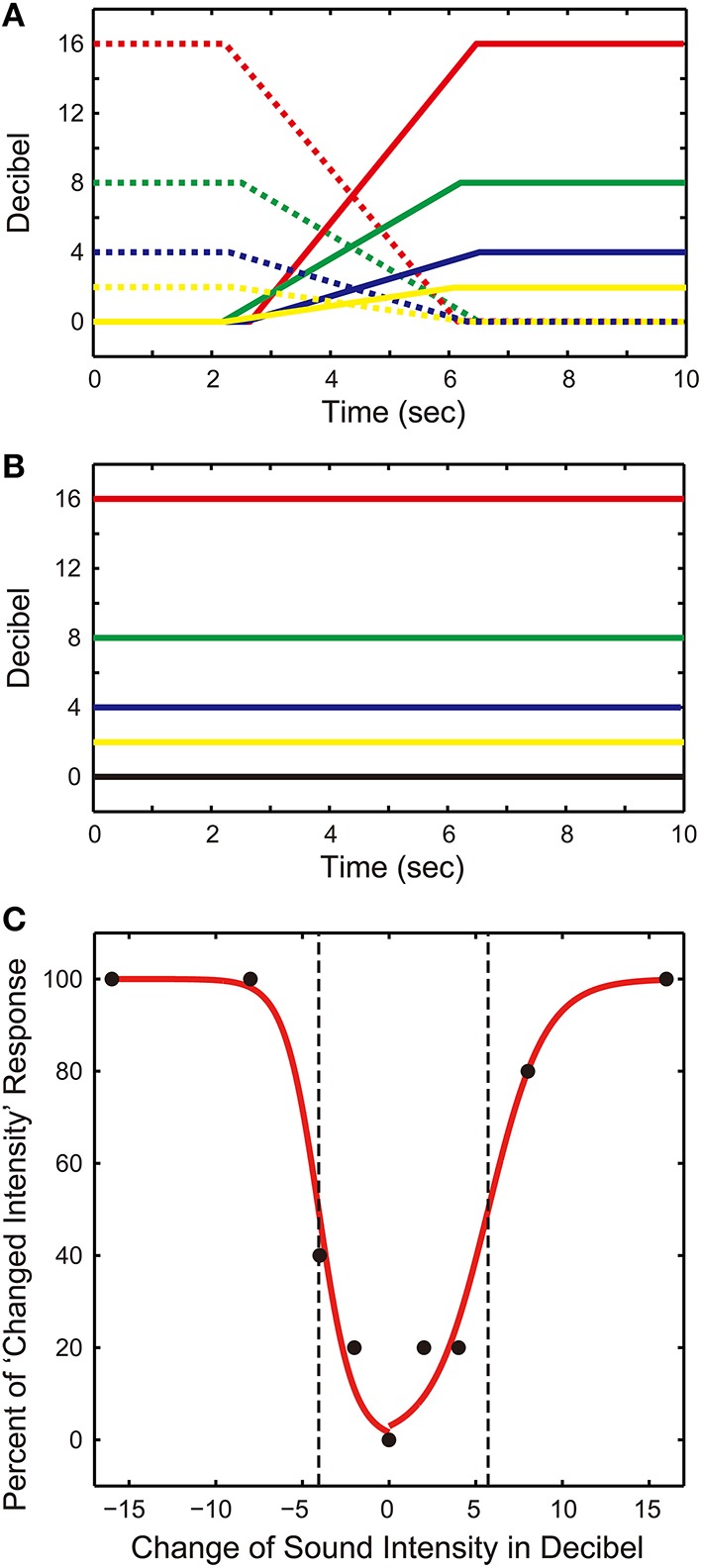
**Hearing test #2. (A)** Patterns of sound stimuli in test trials. The intensity of a 440-Hz pure tone was increased (solid lines) or decreased (dashed lines) in the middle portion of a trial. There were four levels (2, 4, 8, and 16 dB) of sound intensity changes. **(B)** Pattern of sound stimuli in catch trials. The sound intensity was kept either at 0, 2, 4, 8, or 16 dB throughout the trial. **(C)** Psychometric function. Percent of “changed intensity” response was calculated from a participant's responses (filled circles). A binomial logistic regression model was fitted to the data to draw a psychometric function. The perceptual thresholds were estimated with the chance-level (50%) response value (dashed vertical lines).

For this test, participants pressed a switch when they detected a change in sound intensity; they refrained from pressing the switch if there was no change. There were 48 trials in total comprising 40 test trials and 8 catch trials. Test trials involved a gradual change in sound intensity. Across the 40 test trials, there were eight different patterns in which sound intensity changed, with each pattern repeated five times. The eight patterns comprised four levels (e.g., 2, 4, 8, and 16 dBs) and two directions (increase/decrease) of sound intensity change (see Figure 2A). For example, the 440-Hz pure tone is sounded over 10-s while its intensity linearly increased or decreased at a fixed rate over a 4-s period in the mid-portion of the trial. The time point at which the sound intensity changed was jittered randomly between 2.0 and 2.5 s after the beginning of the trial. This prevented participants from anticipating the onset of a sound change.

There were no changes in sound intensity for catch trials; The tone was sounded over 10-s at a constant intensity (see Figure 2B). Four out of eight catch trials maintained the sound intensity at 0 dB while the other four catch trials maintained the intensity at one of the four levels (e.g., 2, 4, 8, or 16 dB). Note that sound intensity was set in decibels relative to the hearing threshold as determined in the first hearing test. Thus, 0 dB represents the hearing threshold and all other levels are calculated relative to this value.

First, participants practiced four trials comprising two test and two catch trials. Next, 48 trials were presented across two blocks of 24 trials with a break between the blocks. Trial order was randomized for each participant. We calculated percent of “changed intensity” response for each individual (see filled black circles in Figure 2C). In the figure, negative values on the horizontal axis denote conditions where sound intensity decreased, while positive values denote conditions where sound intensity increased (re: test trials). A zero value on the horizontal axis denotes no sound intensity change (re: catch trials). We fitted a binomial logistic regression model (“glmfit” function on Matlab with “binomial” and “logit” settings) to the data to draw a psychometric function. The perceptual thresholds were estimated for each participant by using the chance-level (50%) response value (see dashed vertical lines in Figure 2C). We calculated the mean perceptual thresholds for all participants.

### Reaching task

**Figure 3:**
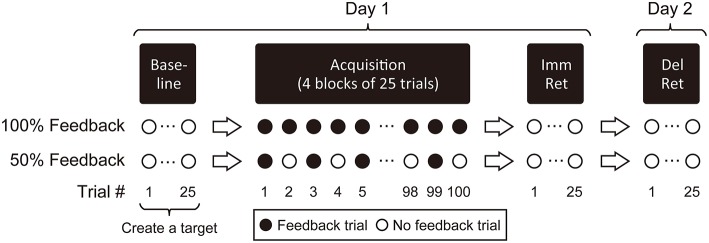
**Reaching task paradigm**. Twenty-five trials of normal reaching movements with no feedback were performed during the baseline phase. These kinematic data were used to create a target joint coordination pattern. Participants then performed four blocks of 25 trials (100 trials) to acquire the target joint coordination pattern (acquisition phase); they received auditory feedback on every (100% feedback) or every other practice trial (50% feedback). Twenty-five no-feedback trials were performed immediately after the acquisition phase (Imm Ret: immediate retention phase on Day 1), and an additional 25 no-feedback trials were performed the next day (Del Ret: delayed retention phase on Day 2).

**Figure 4:**
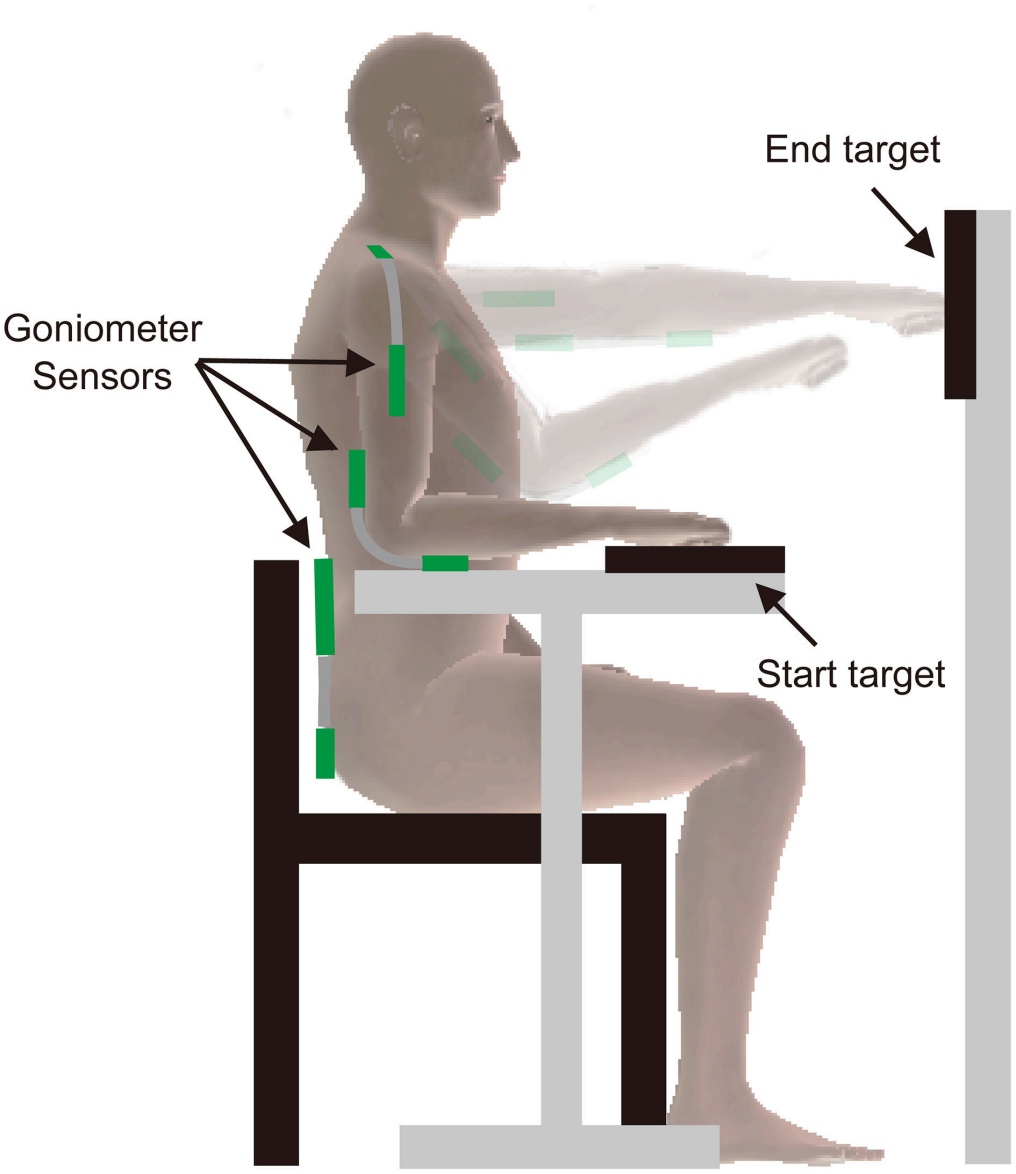
**Setup for reaching task**. Goniometer sensors were attached across the elbow, shoulder, and trunk joints. Participants were seated with their forearm resting on the table. Participants performed a reach by displacing their fist from the start target, reaching toward the end target, hitting it with their fist, and returning to the start target.

**Figure 5:**
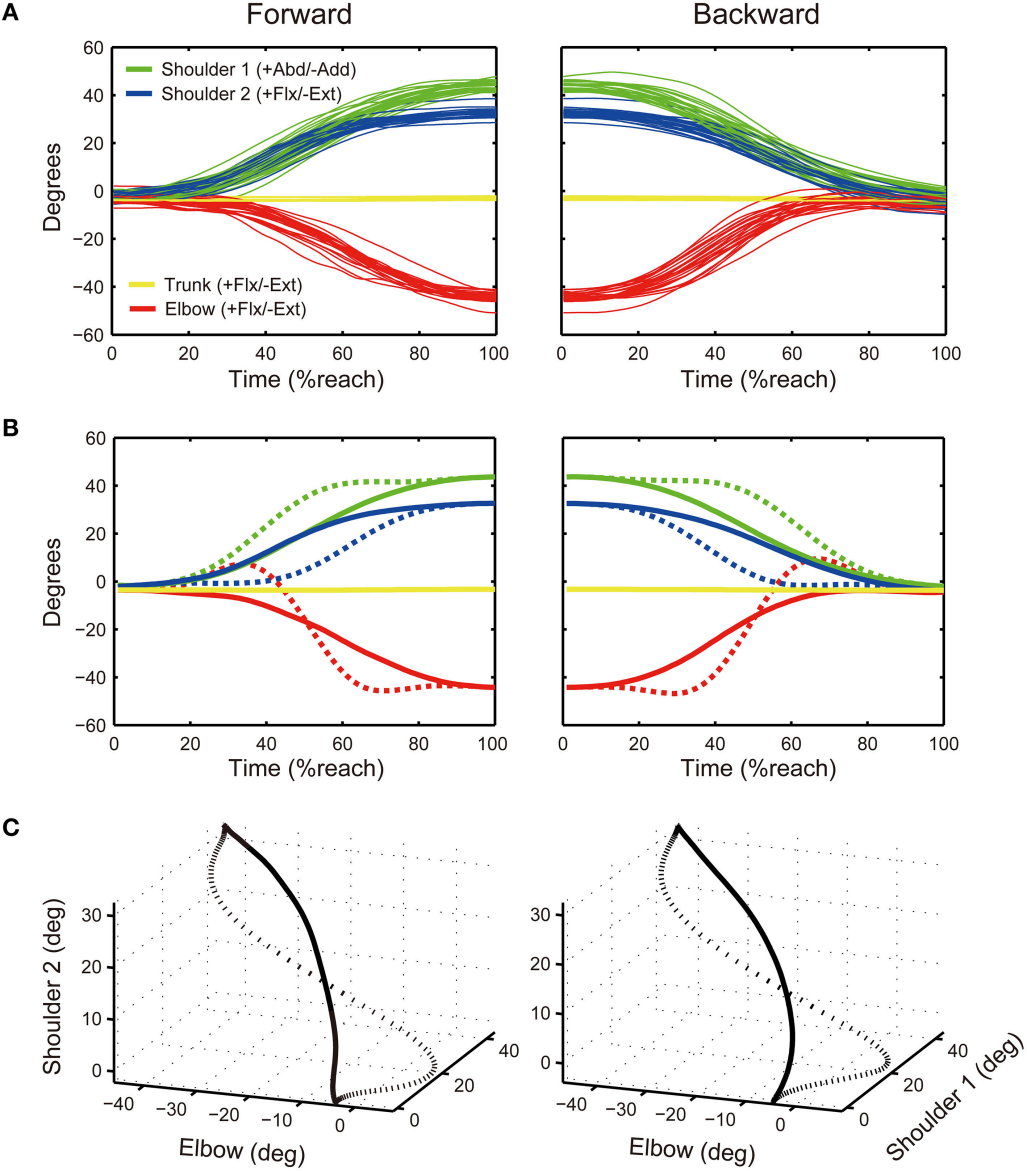
**Processing of baseline data. (A)** A typical example of data recorded during the baseline phase from a participant (right arm, 25 trials). The left panel shows data during the forward reach while the right panel shows data during the backward reach. For the shoulder, a twin-axis goniometer was used that enabled the recording of shoulder abduction/adduction (Abd/Add; Shoulder 1, green line) and shoulder flexion/extension (Flx/Ext; Shoulder 2, blue line). For the elbow and trunk goniometer sensors, a single-axis goniometer was used that recorded elbow flexion/extension (Flx/Ext) and trunk forward flexion and extension (Flx/Ext; see red and yellow lines). **(B)** Averaged time series across the 25 trials (solid lines) and deflected time series (dashed lines). **(C)** Three-dimensional plot consisting of Elbow, Shoulder 1, and Shoulder 2 signals. Solid line denotes the average joint coordination pattern during the baseline, while dashed line denotes the deflected target joint coordination pattern.

The reaching task was performed across two consecutive days (Figure 3). On Day 1, participants first performed 25 trials of baseline reaching (termed as “baseline phase”). Participants were seated with their forearm resting on the table and placed their hand on a start target (Figure 4). Participants reached to an ipsilateral end target in the sagittal plane, using shoulder flexion and elbow extension with no trunk displacement. A novel target joint coordination pattern was then created for each individual based on that person's kinematic data acquired during the baseline phase (see dashed lines in Figure 5 and Section “Creation of Target Joint Coordination Pattern” below). The novel target joint coordination pattern can be described as follows: At the beginning of the reach, participants flexed the elbow and abducted the shoulder. In the middle portion of the reach, they extended the elbow while keeping the shoulder abducted. At the last portion of the reach, they flexed/adducted the shoulder to hit the end target. This novel target joint coordination pattern could be described like a “hook punch” movement in boxing. Importantly, participants were instructed to wear an eye mask while keeping their eyes closed throughout the task to prevent naturally available visual feedback from influencing motor learning.

Participants attempted to learn the novel joint coordination pattern across 100 trials of practice (“acquisition phase”). Participants in the 100% auditory feedback (AF) group received feedback on every trial while those in the 50% AF group received feedback on every other trial (Figure 3). The experimenter informed participants that the tone would become louder as a performed joint coordination pattern deviated from the target joint coordination, while it would become quieter as performance became closer to the target joint coordination (see Section “Error in Joint Coordination Pattern” below). Thus, participants were instructed to minimize the sound intensity to the best of their ability. During the no feedback trials for the 50% AF group, participants were instructed to perform the target joint coordination pattern to the best of their ability (since no feedback was guiding them).

Immediately after the acquisition phase on Day 1, participants performed 25 trials of reaching without feedback to assess immediate retention of the learned joint coordination pattern (“immediate retention phase”). On Day 2, participants performed 25 trials of reaching without feedback to assess delayed retention of the learned joint coordination pattern (“delayed retention phase”).

#### Setup for reaching task

To measure movement kinematics during arm reaching, we used three goniometer sensors (Biometrics Ltd. UK). The sensors were attached using double-sided medical adhesive tape across three joints (elbow, shoulder, and trunk; see Figure 4). The proximal endblock of the elbow goniometer was attached to the arm with its center axis coincident with the center axis of the arm; the distal endblock was attached to the forearm with its center axis coincident with the center axis of the forearm. The proximal endblock of the shoulder goniometer was attached over the belly of the trapezius muscle aligning the distal end of the proximal endblock with the acromion, while the distal endblock was attached to the humerus with its center axis coincident with the center axis of the lateral side of the humerus with the inter-endblock distance of 14 cm. The lower endblock of the trunk goniometer was attached to the lumbar spine with its center axis coincident with the center of the spine aligning the top level of the endblock to the level of L5; the upper endblock was attached to the thoracic spine with its center axis coincident with the center of the spine with the inter-endblock distance of 7 cm. The sensor positions were marked on the skin with a pen to ensure identical placement of goniometer sensors across the 2 days. We used single-axis goniometers to collect data from the elbow (flexion/extension) and trunk (flexion/extension; shown as “Elbow” and “Trunk” in Figure 5A). We used a twin-axis goniometer to record data from the shoulder (abduction/adduction: “Shoulder 1,” flexion/extension: “Shoulder 2”).

The height of the chair was fixed at 46 cm. We adjusted the height of the table for each participant to ensure the forearm was in a comfortable position. The average height of the table was 69.1 ± 2.5 cm (mean ± standard deviation). The start target was embedded into the surface of the table and consisted of an electric switch (1.5 V battery) that recorded the start time of a reach. The end target was placed in front of the participant at arm's length and at shoulder level (with the shoulder at 90° of flexion and elbow at 0° of flexion). The end target also consisted of an electric switch (1.5 V battery) that recorded the time when the end target was hit. The center-to-center distance from the start to the end targets was 40.2 ± 4.5 cm. The start and end targets were aligned in the same sagittal plane, ipsilateral to the reaching arm. The position of the start and end target switches were fixed throughout the reaching task. The distance from the front legs of the chair to the end target was 28.7 ± 5.0 cm. Participants were seated with their initial arm position in 97.4 ± 11.4 degrees of elbow flexion, 31.4 ± 6.6 degrees of shoulder abduction, with the hand closed in a fist, resting on the start target.

Signals of the goniometer sensors were amplified with the K800 amplifier (Biometrics Ltd., UK). Signals of the electric switches and goniometer sensors were synchronized and converted from analog to digital at a frequency of 200 Hz with the AIO board, and recorded on a personal computer with a custom written program in C++. Goniometer data was low-pass filtered offline using a 4th order Butterworth filter with a cut off frequency of 10 Hz by custom written scripts in Matlab software (Mathworks, USA).

#### Creation of target joint coordination pattern

The joint coordination pattern for reaching during the baseline phase was used to create a novel target joint coordination pattern to be learned by participants. A typical data set from the 25 baseline trials of a participant is shown in Figure 5A. The left panel shows data during the forward portion of the reach (start to end target) while the right panel shows data during the backward portion of the reach (end to start target). The x-axis represents time, normalized as percentage of reach (% reach) based on duration recorded from the start and end target switches. The average of 25 trials for each joint is shown as solid lines in Figure 5B.

The average joint coordination pattern is represented as a trajectory in three-dimensional joint coordination space, consisting of the averaged Elbow, Shoulder 1, and Shoulder 2 signals (average of 25 trials for each joint, see solid black line in Figure 5C). The Trunk signal is not included since no participant moved the trunk.

To create the novel target joint coordination pattern, we “deflected” the Elbow (E), Shoulder 1 (S1), and Shoulder 2(S2) signals (see dashed lines in Figures 5B,C; see also the Supplementary Material for details on how to deflect the trajectory). The idea to deflect the movement pattern was based on a previous reaching study that deflected each individual's baseline trajectory to create a novel target reaching pattern (Wu et al., 2014). We applied this idea because it allowed us to create a novel and unfamiliar upper limb coordination pattern for each individual to learn using augmented auditory feedback.

#### Error in joint coordination pattern

**Figure 6:**
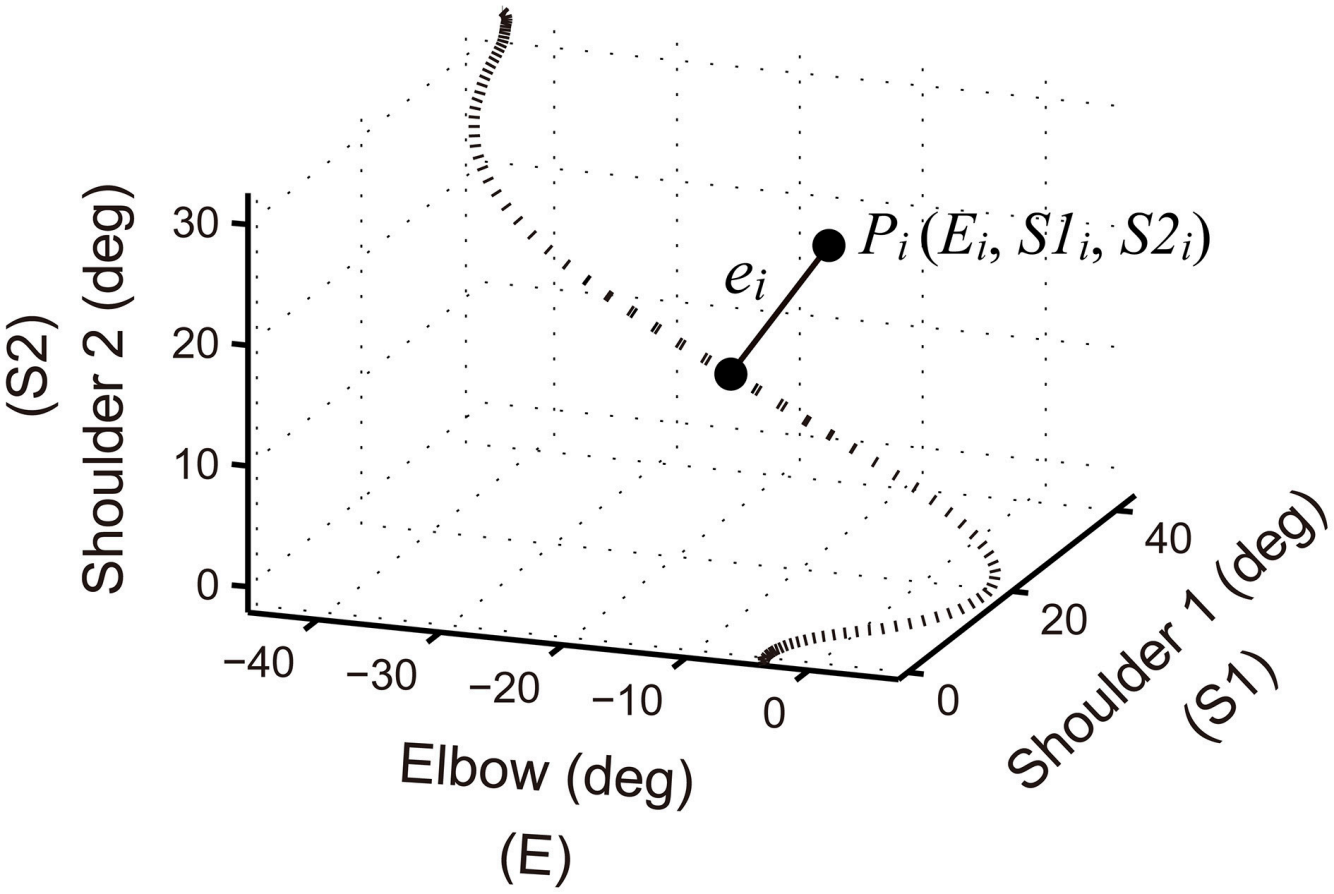
**Schematics of joint coordination error**. A performed joint coordination pattern at the *i*-th sampled time frame can be drawn as a point (*P*_*i*_) in the three-dimensional joint coordination space. The pattern is derived from the Elbow (E), Shoulder 1 (S1), and Shoulder 2 (S2) signals measured with the goniometer sensors. The dashed line denotes the target joint coordination pattern. An error at the *i*-th sampled time frame (*e*_*i*_) can be visualized as the minimum distance from *P*_*i*_ to the target trajectory. Auditory feedback was created by changing the sound intensity of a pure tone in proportion to the amount of error.

To create concurrent KP auditory feedback, data from the goniometer sensors were processed every 10 ms in the C++ program. To assess how a performed joint coordination pattern deviates from the target joint coordination (defined from the deflection of the baseline reach), we define an error at *i*-th sampled time frame (*e*_*i*_) as,

(1) $$\begin{array}{l} e_{i}=\sqrt{{\left(E_{i}-E_{j}\right)}^{2}+{\left(S1_{i}-S1_{j}\right)}^{2}+{\left(S2_{i}-S2_{j}\right)}^{2}}; \end{array}$$

where *E*_*i*_, *S1*_*i*_, and *S2*_*i*_ are *i*-th sampled time frame of performed joint angles measured by the goniometer sensors at the elbow and shoulder. For example, a performed joint coordination at the *i*-th sampled time frame can be drawn as a point (*P*_*i*_) in the three-dimensional joint coordination space (Figure 6). *E*_*j*_, *S1*_*j*_, and *S2*_*j*_ are *j*-th sampled time frame of the target joint angles where the distance from *P*_*i*_ to the target trajectory becomes minimum. Thus, an error (*e*_*i*_) can be drawn as the minimum distance from *P*_*i*_ to the target trajectory (Figure 6). The intensity of the feedback sound at the *i*-th time frame (*I*_*i*_) is then set in decibels to be twice as large as the amount of error in degrees;

(2) $$\begin{array}{l} I_{i}=2\times e_{i} \end{array}$$

Thus, sound intensity in decibels was set as zero (*I*_*i*_ = 0) if a performed joint coordination perfectly matched the target (*e*_*i*_ = 0), while for example, a participant hears a 20 dB tone if the error is 10° from the target. Here, zero decibels correspond to the hearing threshold as determined in hearing test #1.

#### Measures to assess joint coordination pattern

To assess the degree to which participants achieved the target joint coordination pattern, we calculated the root-mean squared error (RMSE) between the performed and target trajectories in the three-dimensional joint coordination space:

(3) $$\begin{array}{l} RMSE=\frac{1}{n}\sum_{i=1}^{n}e_{i}; \end{array}$$

where *e*_*i*_ is the error of joint coordination at the *i*-th sampled time frame (see Figure 6) and *n* is the total number of data points. Note, that root of the squared error (RSE) was already calculated in Equation (1) and therefore the mean over the time points (RMSE) was calculated in Equation (3).

**Figure 7:**
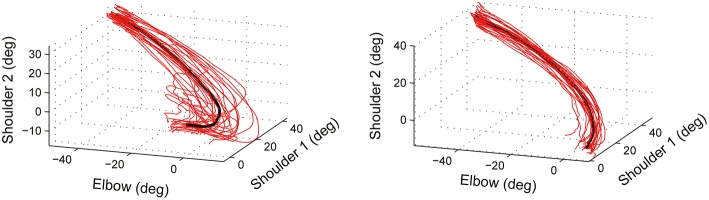
**Schematics of variable error (VE)**. Data from 25 trials of reaching are plotted in each of the left and right panels (red lines). Black line denotes the average across the 25 trials. The variable error (VE) was calculated as the RMSE between a performed trajectory (the red line) and the mean trajectory (the black line). The VE is larger in the left example compared to the right one.

The RMSE assesses the degree to which the performed joint coordination pattern deviates from the target joint coordination pattern. To assess the consistency of joint coordination across trials, we calculated the variable error (VE; Schmidt and Lee, 2011). The calculation of VE is similar to that of RMSE but differs in the reference trajectory used to quantify the error. To calculate the RMSE, the target joint coordination pattern was used as the reference trajectory. On the other hand, the mean joint coordination trajectory across the 25 trials in a block was used as the reference trajectory to evaluate the error in the VE measure (see Figure 7). In Figure 7, an example of inconsistent and consistent joint coordination patterns is shown in the left and right panels, respectively. The data from 25 trials of reaching are plotted in each of the left and right panels. The black line in the figure shows the average across the 25 trials. (Note, that the black line is not the target trajectory.) For each trial, the VE was calculated as the RMSE between a performed trajectory (a red line) and the mean trajectory (the black line). The VE is larger in the left example compared to the right one.

The RMSE and VE were calculated from data acquired between 20 and 80% of the reach where the main deflection was made to create the target trajectory. For each individual, the RMSE and VE were calculated for each trial during the acquisition and retention phases then averaged across 25 trials in each block. Note that the RMSE and VE were calculated for acquisition and retention data but not for baseline data. This was because participants had no “target” during baseline since they performed 25 trials of normal reaching during this phase.

### Statistics

Consistent with previous studies (Winstein and Schmidt, 1990; Nicholson and Schmidt, 1991; Vander Linden et al., 1993; Tal, 1995; Wulf et al., 1998, 2010; Park et al., 2000), acquisition and retention data were analyzed separately. For the acquisition phase, the RMSE and VE were subjected to a two-way repeated-measures analysis of variance (ANOVA) with the within-participant factor of Block (Block 1, 2, 3, and 4) and the between-participant factor of Group (100 and 50% AF groups). For the retention phase, the RMSE and VE were subjected to a two-way repeated-measures ANOVA with the within-participant factor of Day (immediate retention on Day 1 and delayed retention on Day 2) and the between-participant factor of Group (100 and 50% AF groups). The perceptual threshold to detect a change in sound intensity (measured in hearing test #2) was subjected to an independent-samples *t*-test to compare thresholds between the 100 and 50% AF groups. We used the Mann-Whitney *U* tests to compare the 100 and 50% AF groups on age, handedness, number of years of education, and number of years of musical training. Significance was set at *P* < 0.05 (two-tailed) for all statistical tests.

## Results

### Demographics

There was no Significant Difference in Age, Handedness, Number of Years of Education, and Number of Years of Musical Training Between the Two Groups (*P* > 0.143, see Table 1).

### Perceptual threshold

The perceptual thresholds for the detection of changes in sound intensity (as measured in hearing test #2) are summarized in Table 2. There was no significant difference in perceptual thresholds between the two groups (*P* > 0.35).

**Table 2:** **Hearing test #2: perceptual threshold to detect a change in sound intensity (decibels)**.

**Group**	**Mean (*****SD*****)**		***T*_18_**	***P*-values**
	**50% (*n* = 10)**	**100% (*n* = 10)**		
Perceptual threshold (Intensity decreased)	5.2 (4.2)	5.9 (3.6)	0.396	0.696
Perceptual threshold (Intensity increased)	6.3 (2.7)	7.7 (3.8)	0.970	0.345
Perceptual threshold (Mean)	5.7 (2.9)	6.8 (3.5)	0.736	0.471

*Associated p-values for results of the independent-samples t-tests comparing 50 and 100% auditory feedback groups*.

### A typical joint coordination pattern during acquisition and retention phases

**Figure 8:**
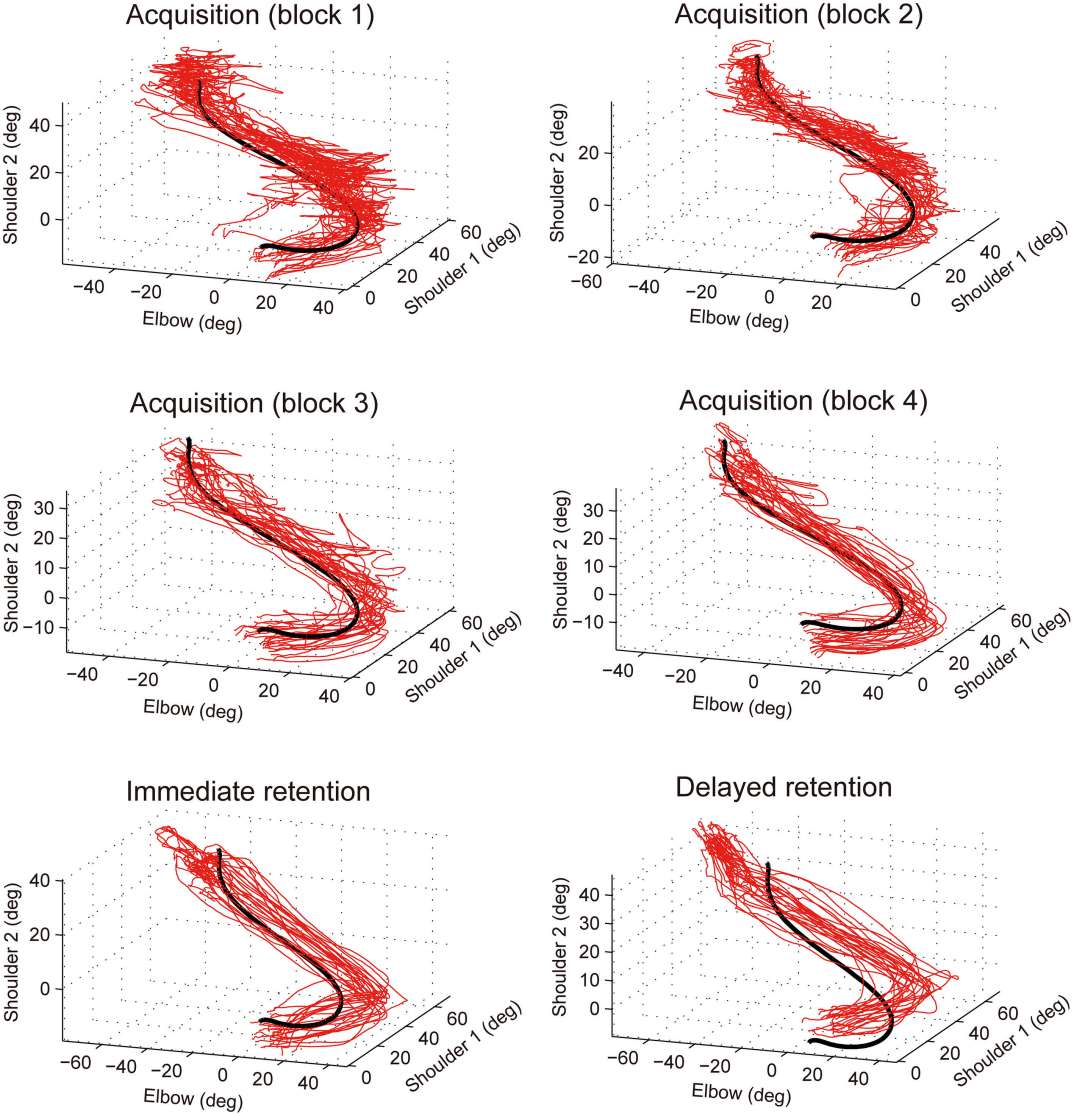
**Examples of joint coordination patterns in acquisition and retention phases**. Data from 25 trials representing the forward reach from a participant in the 100% auditory feedback group. Red thin lines show performed joint coordination patterns while black solid lines show the target joint coordination pattern.

A typical example of the performed elbow and shoulder joint coordination pattern for the forward portion of the reach, during acquisition, and retention phases are shown in Figure 8. The RMSE and VE become smaller over the course of practice blocks during the acquisition phase. That is, the performed trajectories (red lines) become closer to the target (black line) and less variable. The RMSE at the delayed retention phase was larger than that at the immediate retention phase. That is, the performed trajectories (red lines) deviated more from the target (black) compared to those at the immediate retention phase.

### Acquisition phase

**Figure 9:**
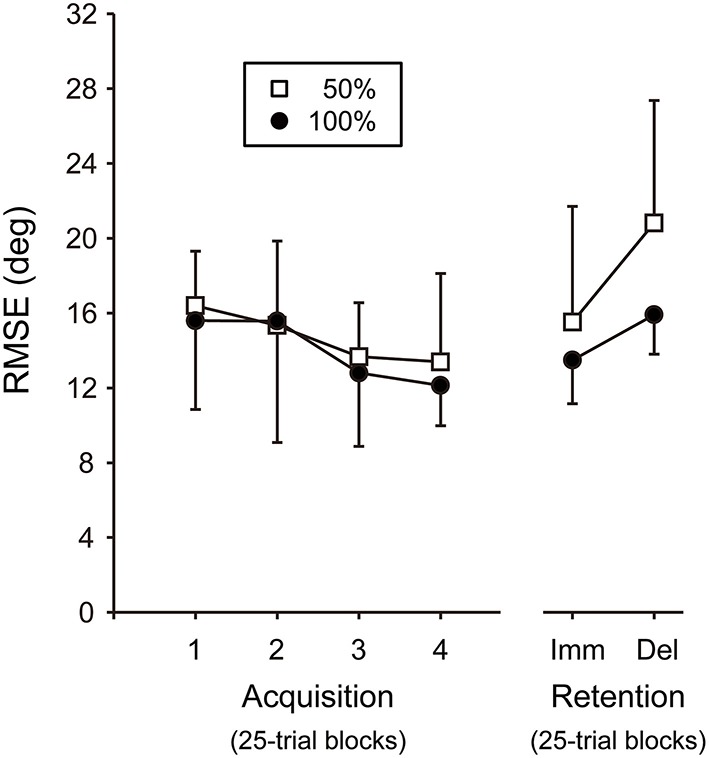
**Root mean squared error (RMSE)**. Imm, immediate. Del, delayed. The 100 and 50% auditory feedback groups are denoted by filled circles and open squares, respectively. The error bar denotes standard deviation across participants.

For the RMSE, there was no significant interaction between the Block and Group factors in the two-way ANOVA [*F*_(3, 54)_ = 0.31, *P* = 0.82, η^2^ = 0.02]. The main effect of Block was significant [*F*_(3, 54)_ = 7.92, *P* < 0.001, η^2^ = 0.31] whereas, that of Group was not [*F*_(1, 18)_ = 0.17, *P* = 0.69, η^2^ = 0.01], showing that both 100 and 50% groups reduced the joint coordination error relative to the target across practice in the acquisition phase (see Figure 9).

**Figure 10:**
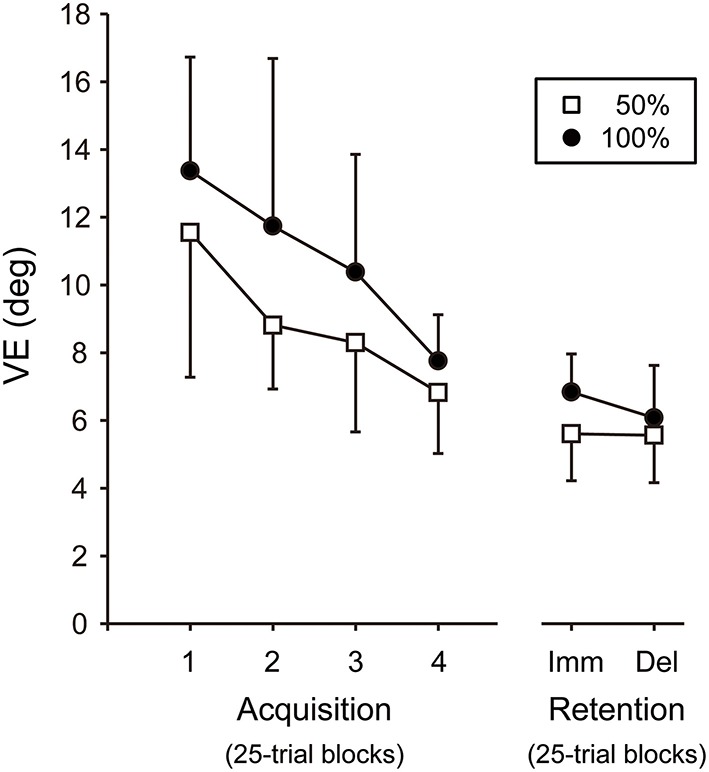
**Variable error (VE)**. Imm, immediate. Del, delayed. The 100 and 50% auditory feedback groups are denoted by filled circles and open squares, respectively. The error bar denotes standard deviation across participants.

For the VE, there was no significant interaction between the Block and Group factors in the two-way ANOVA [*F*_(3, 54)_ = 0.47, *P* = 0.70, η^2^ = 0.03]. The main effect of Block was significant [*F*_(3, 54)_ = 12.80, *P* < 0.001, η^2^ = 0.42] whereas, that of Group was not [*F*_(1, 18)_ = 3.90, *P* = 0.06, η^2^ = 0.18], showing that the VE became smaller in both 100 and 50% groups across practice in the acquisition phase (see Figure 10). Taken together, both RMSE and VE were significantly reduced over the course of training in both 100 and 50% feedback groups, suggesting that auditory feedback guided the joint coordination pattern to the target with less variability during the acquisition phase.

### Retention phase

For the RMSE, there was no significant interaction between the Day and Group factors in the two-way ANOVA [*F*_(1, 18)_ = 0.99, *P* = 0.33, η^2^ = 0.05] (Figure 9). The main effect of Day was significant [*F*_(1, 18)_ = 7.18, *P* < 0.05, η^2^ = 0.29], showing that RMSE was smaller at the immediate retention phase compared with the delayed retention phase. The main effect of Group was also significant [*F*_(1, 18)_ = 4.86, *P* < 0.05, η^2^ = 0.21], showing that the RMSE of the 100% AF group was significantly smaller than that of the 50% AF group at both retention phases.

For the VE, there was no significant interaction between the Day and Group factors in the two-way ANOVA [*F*_(1, 18)_ = 1.15, *P* = 0.30, η^2^ = 0.06] (Figure 10). No main effect of Day nor Group was found in the ANOVA [Day: *F*_(1, 18)_ = 1.40, *P* = 0.25, η^2^ = 0.07; Group: *F*_(1, 18)_ = 2.89, *P* = 0.11, η^2^ = 0.14, respectively]. Taken together, the 100% AF group showed smaller RMSE than the 50% AF group while VE was comparable between the groups at both retention phases.

## Discussion

The purpose of this study was to test the guidance hypothesis (Salmoni et al., 1984; Schmidt et al., 1989) in the context of learning a novel joint coordination pattern with concurrent KP auditory feedback. According to the guidance hypothesis (Salmoni et al., 1984; Schmidt et al., 1989), we predicted the following: First, the 50% AF group would show better retention of learned joint coordination patterns after the removal of auditory feedback, compared to the 100% AF group. Second, the 50% AF group would show less variable joint coordination patterns than the 100% AF group. Contrary to the first prediction, the 100% AF group showed better retention of the learned joint coordination pattern (i.e., smaller RMSE) at both immediate and delayed retention phases, than the 50% AF group. Contrary to the second prediction, there was no significant difference in VE between the 50 and 100% AF groups for either acquisition or retention phases. Thus, the guidance hypothesis was not supported in this study using our specific type of sonified feedback manipulation. Our results suggest that concurrent KP auditory feedback facilitates learning of a novel joint coordination pattern when the feedback is presented more frequently.

### Why more is better

In our study, the 100% AF group showed better retention of the learned joint coordination pattern compared to the 50% AF group. These findings are in contrast with those from prior research that showed better retention of performance when the skill was learned with a reduced frequency of feedback (Winstein and Schmidt, 1990; Nicholson and Schmidt, 1991; Vander Linden et al., 1993; Park et al., 2000).

We suggest that task complexity could be a main reason why more feedback led to better retention in this study. While some studies support the guidance hypothesis (Winstein and Schmidt, 1990; Nicholson and Schmidt, 1991; Vander Linden et al., 1993; Park et al., 2000), others do not (Wulf et al., 1998, 2010). Studies that do not support the guidance hypothesis showed that frequent augmented feedback resulted in better retention of the learned skill. These findings are consistent with our results. Wulf and Shea (2002) pointed out in their review that studies supporting the guidance hypothesis used relatively simple tasks seen in typical laboratory settings (e.g., the lever patterning task in the study by Winstein and Schmidt (1990). In contrast, studies that do not support the guidance hypothesis use more complex tasks such as those that mimic real-life learning situations (e.g., the ski simulator task in the study by Wulf et al. (1998) and the bimanual soccer throw-in task in the study by Wulf et al. (2010). The reaching task in the present study could be regarded as relatively complex. In fact, some participants in this study reported that the task was very demanding. Thus, there may be an interaction between task complexity and feedback frequency (Wulf et al., 1998; Wulf and Shea, 2002). The learning of simple motor skills may benefit from a reduced frequency of feedback while the learning of more complex motor skills may benefit from a higher frequency of feedback. Taken together, task complexity could explain why more feedback led to better retention.

One might assume that the modality of feedback could also be a factor that explains the discrepancy. To test the guidance hypothesis, previous studies used augmented visual feedback (Winstein and Schmidt, 1990; Nicholson and Schmidt, 1991; Vander Linden et al., 1993; Swinnen et al., 1997; Wulf et al., 1998; Park et al., 2000). To our knowledge, a limited number of studies used augmented auditory feedback and showed that it did not deteriorate performance of the learned skill at retention (Ronsse et al., 2011; Sigrist et al., 2015). Interestingly, the provision of visual feedback did negatively affect performance (Ronsse et al., 2011). Moreover, audiovisual feedback better facilitated the learning of a target rowing velocity compared to visuohaptic feedback (Sigrist et al., 2015). Thus, the use of augmented auditory feedback (in contrast to other modalities of feedback) may also be one reason why more feedback led to better retention in this study. However, at this point, it is not clear how the modality of feedback and frequency of feedback interact. Future studies are needed to clarify this issue.

### Potential confounders: Perception and age

First, one might assume that the difference between the 100 and 50% AF groups could be simply attributed to a difference in auditory perception. However, our results from hearing test #2 rule out this possibility. There was no significant difference between the two groups in their perceptual thresholds to detect changes in sound intensity. In addition, there was no significant difference in the number of years of musical training, showing that musical background was also comparable between groups. Thus, the observed group difference in this study could not be attributed to a difference in auditory perceptual capability.

Second, one might assume that the age of participants could be a confounder. The mean age of participants in this study (i.e., 34.0 years of age) was relatively higher compared with those of previous studies that showed the advantage of reduced frequency of feedback for motor learning (Winstein and Schmidt, 1990; Nicholson and Schmidt, 1991; Vander Linden et al., 1993; Park et al., 2000; these studies tested mostly undergraduate students as participants). Therefore, one might think that more feedback would lead to better skill retention in an older population because older adults may demonstrate slower motor learning (Fernandez-Ruiz et al., 2000) and require more information to help them learn. To test this possibility, we performed an additional analysis to investigate the relationship between age and RMSE at retention (i.e., averaged RMSE across the immediate and delayed retention phases). We found no significant correlation for the 100% AF group (Spearman's ρ = −0.55, *P* = 0.10) or for the 50% AF group (ρ = −0.10, *P* = 0.79). Thus, there was no significant relationship between age and retention of performance in this study. In addition, previous studies show that both younger and older adults process feedback similarly (Swanson and Lee, 1992; Wishart and Lee, 1997), and benefit from reduced frequency of feedback to learn (Tal, 1995). This suggests that an older population does not necessarily need more feedback to learn a skill. Accordingly, age may not be a factor to explain why more feedback led to better retention in this study.

### The role of movement variability in motor learning

During the acquisition phase, the 100% AF group showed a trend toward increased variability in the joint coordination pattern (i.e., larger VE) compared to the 50% AF group (*P* = 0.06). The guidance hypothesis views increased variability as a negative outcome, defining it as maladaptive short-term corrections (Schmidt, 1991). In contrast, more recent motor-control studies view movement variability as an essential ingredient that facilitate motor learning (Herzfeld and Shadmehr, 2014; Wu et al., 2014). Given that the 100% AF group showed better retention of the novel joint coordination pattern, the tendency toward an increased variability in the joint coordination pattern observed during acquisition may be viewed as adaptive (and not maladaptive) corrections in this study. That is, the tendency for increased movement variability in the 100% AF group may have a functional role, helping the learner adapt to a new situation. Specifically, the reaching task in this study required participants to map changes in sound intensity onto changes in joint coordination patterns. Therefore, if joint coordination patterns were more variable, participants would be able to acquire more information about the auditory-motor mapping. This might then help the learner to develop a more advanced internal model, which may lead to better retention of the learned joint coordination pattern.

If the above assumption is correct, there should be a tight relationship between VE during the acquisition phase and RMSE at the retention phase. A learner, who experiences more variable joint coordination patterns during the acquisition phase, would show better retention of the target joint coordination pattern at the immediate and delayed retention phases. Thus, a significant correlation between the two measures is expected. We therefore performed an analysis to investigate the relationship between the VE averaged across the four acquisition blocks and the mean RMSE across the immediate and delayed retention phases. However, there was no significant correlation for the 100% AF group (Pearson's *r* = −0.31, *P* = 0.39) or for the 50% AF group (*r* = −0.55, *P* = 0.10). Thus, we cannot make a strong case regarding a potential relationship between increased movement variability and learning. Future studies are needed to clarify the role of movement variability for the learning of a novel joint coordination pattern with augmented auditory feedback.

## Conclusion

Our study demonstrates that concurrent KP auditory feedback may facilitate the learning of a novel upper-limb joint coordination pattern when it is provided during all practice trials as opposed to during half of the trials. Our finding will help us better understand how to facilitate the (re)learning of organized joint coordination patterns with auditory feedback during motor skill acquisition and rehabilitation.

## Author contributions

SF, TL, and JC conceived and designed the study. SF and TL performed the experiment. SF analyzed the data. SF, TL, and JC interpreted the data and wrote the paper.

### Conflict of interest statement

The authors declare that the research was conducted in the absence of any commercial or financial relationships that could be construed as a potential conflict of interest.
