# Supplementary material for: More Feedback Is Better than Less: Learning a Novel Upper Limb Joint Coordination Pattern with Augmented Auditory Feedback
Source: Front Neurosci. 2016 Jun 6;10:251. doi: 10.3389/fnins.2016.00251 (PMC4893479; doi:10.3389/fnins.2016.00251)
Supplement: Supplementary file 1 [file Presentation1.PDF]

Supplementary Material

More feedback is better than less: Learning a novel upper limb joint coordination pattern with augmented auditory feedback

Shinya Fujii, Tea Lulic, Joyce L. Chen*

*** Correspondence:** Corresponding Author: j.chen@sri.utoronto.ca

Supplementary Methods

Deflection function

To create a novel joint coordination during arm reaching, we 'deflected' the Elbow, Shoulder 1, and Shoulder 2 signals to create a target joint coordination pattern. We did not deflect the trunk signals since participants did not move the trunk but instead, coordinated their elbow and shoulder joints during the baseline reaching. The deflection was made based on a previous arm-reaching study that deflected each individual's baseline trajectory to create a novel target reaching pattern (Wu et al., 2014). We applied this idea because it allowed us to create an 'unfamiliar' upper limb coordination pattern for each individual, which allowed us to test whether augmented feedback would be able to guide a novel upper limb coordination pattern. The deflection was made by using sine functions, which was multiplied by a Blackman window (**Supplementary Figure 1**). The sine function had two pi periods for the elbow data (top panels) and had one pi period for the shoulder data (middle and low panels). The amplitudes of the sine function were 15 degrees. We used the Blackman window to keep the baseline joint coordination pattern at the beginning and end of the reach, which ensured participants could keep their original posture when touching the start and end targets.

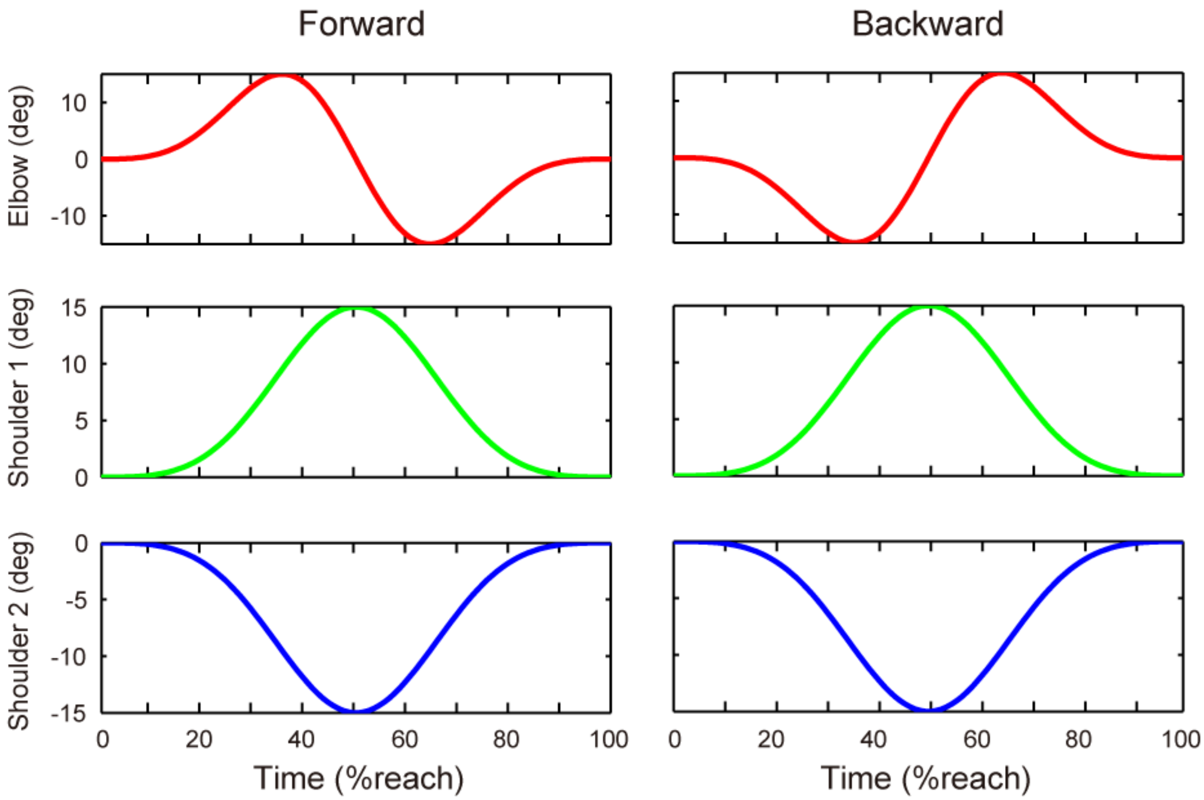

Supplementary Figure 1: Deflection functions for elbow (top) and shoulder (middle and low) goniometer signals to create a target joint-coordination pattern. Left panel shows the function for forward reaching movements while right panel shows that for backward reaching movements.

Supplementary References

Wu, H.G., Miyamoto, Y.R., Gonzalez Castro, L.N., Olveczky, B.P., and Smith, M.A. (2014). Temporal structure of motor variability is dynamically regulated and predicts motor learning ability. *Nat Neurosci* 17**,** 312-321. doi: 10.1038/nn.3616
